# Bulimia nervosa and borderline personality disorder - case report and literature review

**DOI:** 10.1192/j.eurpsy.2022.407

**Published:** 2022-09-01

**Authors:** A. Borges, D. Machado

**Affiliations:** Centro Hospitalar de Leiria, Serviço De Psiquiatria E Saúde Mental, Leiria, Portugal

**Keywords:** Eating Disorders, Borderline Personality, Bulimia nervosa

## Abstract

**Introduction:**

Bulimia Nervosa (BN) is a debilitating eating disorder characterized by binging and purging episodes generally accompanied by excessive concern with body weight and shape as well as body image disturbance. BN and Borderline Personality Disorder (BPD) may co-occur. In fact, studies estimate that one quarter to one third of patients with BN also meet criteria for BPD. However not much is known about the relationship between these two diseases. Nevertheless, the high comorbidity rate might not be surprising as both BN and BPD may share interacting aetiologies and common core symptoms such as impulsivity and emotional instability. So far, only very little is known about the clinical presentation of patients with both BN and BPD and their response to treatment.

**Objectives:**

Literature review on BN and comorbid BPD. An illustrative clinical case is presented.

**Methods:**

Case report and non-systematic review of the literature - sources obtained through search on Pubmed.gov database.

**Results:**

Female, 19-year-old, student, lived with her mother and stepfather. Developed a poor relationship with her body image due to dental problems during high school. The patient started to binge eat, exhibit compensatory behaviors, restrictive eating pattern, body dissatisfaction and emotional instability while maintaining a normal BMI. Over the last year, she started a self-destructive behavior with slight improvement of BN symptoms.

**Conclusions:**

Special attention should be given to patients suffering from BN and comorbid BPD as they present greater risk of recurrent suicide attempts and non-suicidal self-injury, as well as lower rates of remission. Early interventions that target impulsivity and problematic eating behavior may mitigate risk of future borderline personality features.

**Disclosure:**

No significant relationships.

